# Relative expression of proprotein convertases in rat ovaries during pregnancy

**DOI:** 10.1186/1757-2215-6-91

**Published:** 2013-12-11

**Authors:** Simon CM Kwok, Damayanti Chakraborty, Michael J Soares, Guoli Dai

**Affiliations:** 1 ORTD, Albert Einstein Medical Center, 5501 Old York Road, Philadelphia, PA 19141-3098, USA; 2 Institute for Reproductive Health and Regenerative Medicine, University of Kansas Medical Center, Kansas City, Kansas, USA; 3 Department of Biology, Indiana University-Purdue University Indianapolis, Indianapolis, IN, USA

**Keywords:** Prohormone convertase, Gene expression, Posttranslational processing

## Abstract

**Background:**

Proprotein convertases are a family of serine proteinases that are related to bacterial subtilisin and yeast kexin. They are involved in posttranslational processing of the precursors of a vast number of cellular proteins. With the exception of PC1/3, the relative expression levels of the proprotein convertases in the ovary during pregnancy have not been reported. The purpose of this study is to determine by real-time PCR the relative expression levels of all nine proprotein convertases in rat ovaries during pregnancy and at 3 days postpartum.

**Methods:**

RNA was extracted from ovaries at Day 0, 4, 9, 11, 13, 15, 18, and 20 of pregnancy as well as 3 days postpartum. Relative expression levels of Pcsk1, Pcsk2, Furin, Pcsk4, Pcsk5, Pcsk6, Pcsk7, Mbtps1 and Pcsk9 were determined with real-time PCR. Results were reported as fold-change over the level at Day 0 of pregnancy.

**Results:**

Results showed that *Pcsk1* and *Pcsk6* were upregulated as gestation advanced, in parallel with an observed increase in relaxin transcript. *Pcsk2* showed downregulation as gestation advanced, while *Pcsk5* showed relatively higher levels in early pregnancy and postpartum, but lower level in mid-pregnancy. On the other hand, *Furin, Pcsk4, Pcsk7, Mbtps1 and Pcsk9* showed little change of expression throughout gestation.

**Conclusion:**

PC1/3 (PCSK1) and PACE4 (PCSK6) may play an important role in proprotein processing in the ovary during late pregnancy.

## Background

Proprotein convertases are a family of serine proteinases that are related to bacterial subtilisin and yeast kexin. They are involved in posttranslational processing of a vast number of cellular proteins leading to their activation and sometimes inactivation. There are nine members identified so far: they are PC1/3, PC2, Furin, PC4, PC5/6, PACE4, PC7, SKI-1/S1P, and PCSK9 (NARC-1) [1]. Their genes are named *Pcsk1, Pcsk2, Furin, Pcsk4, Pcsk5, Pcsk6, Pcsk7, Mbtps1, and Pcsk9.* The first seven members process precursor proteins at single or paired basic amino acids with the motif of (R/K)X_n_(R/K)↓ (where R = arginine, K = lysine, X = any amino acid). They share a cleavage redundancy towards numerous substrates, such as protein hormones, receptors, adhesion molecules and metalloproteinases. The eighth member, SKI-1/S1P, cleaves membrane-bound transcription factors and N-acetylglucosamine-1-phosphotransferase at the motif of RX(L/V/I)X↓ (where L = leucine, V = valine, I = isoleucine), and the last member, PCSK9, cleaves itself at VFAQ^152^↓ (valine-phenylalanine-alanine-glutamine^152^) sequence [2].

Of these nine proprotein convertases, Furin, PC5/6, PACE4, PC7, and SKI-1/S1P are ubiquitously expressed [3-7]. Expression of PC1/3 and PC2 is restricted to neuroendocrine tissues [8]. PC4 is expressed primarily in male germ cells, although it is also expressed in ovary and placenta [9]. PCSK9 (NARC-1) is expressed in adult liver, small intestine and kidney [10]. These proprotein convertases have been implicated to play an important role in follicle development and ovulation. PC5/6 was upregulated in rat ovarian follicles by gonadotropins and may be involved in the processing of precursors of transforming growth factor beta (TGFβ) and matrix metalloproteinases [11]. Both PC5/6 and inhibin were upregulated during follicle development in mouse ovary, suggesting that PC5/6 may be involved in inhibin subunit processing [12]. PACE4 (PCSK6) expression in preantral granulosa cells was upregulated by follicle-stimulating hormone (FSH), but was suppressed by factors secreted by full-grown oocytes from antral follicles [13]. The change of PACE4 expression may suggest a change of mechanism involved in the processing of the precursors of the TGFβ family. Using P*csk*6-knockout mice, Mujoomdar and co-workers [14] showed that PACE4 played an important role in maintaining normal cellular and tissue homeostasis in the ovary. Expression of P*csk*6 in human granulosa cells and oocytes was suppressed by bone morphogenetic proteins, suggesting that it was subjected to bone morphogenetic protein negative feedback [15]. Furin was upregulated in rat ovary treated with gonadotropin, whereas both matrix metalloproteinase 2 (MMP2) activation and oocyte release were decreased after treatment with furin inhibitor [16]. These results suggest a role of furin in the breakdown of follicular wall during ovulation. Furin may also be involved in proliferation of granulosa cells, since knockdown of furin expression with furin siRNA decreased proliferation of granulosa cells [17]. Expression of these proprotein convertases in the ovary during gestation has not been well studied. Our laboratory has cloned and characterized multiple PC1/3 transcripts from porcine ovary [18]. In situ hybridization showed that PC1/3 and relaxin transcripts were colocalized in large luteal cells of porcine ovary and levels of both transcripts increased as gestation advanced [19]. This suggests that PC1/3 may be involved in posttranslational processing of prorelaxin. The purpose of the present study is to investigate the relative expression levels of all nine proprotein convertases in rat ovaries during gestation using real-time PCR.

## Methods

### Animals and tissue preparation

Holtzman Sprague-Dawley rats were obtained from Harlan Laboratories (Indianapolis, IN). The animals were housed in an environmentally controlled facility with lights on from 0600 to 2000 hr and allowed free access to food and water. Timed pregnancies were obtained by housing female rats with male rats and examining vaginal smears daily during the cohabitation. The presence of a copulatory plug or sperm in the vaginal smear was designated Day 0 of pregnancy. Rats were sacrificed on Day 0, 4, 9, 11, 13, 15, 18, 20 of pregnancy as well as 3 days postpartum, and ovaries were quickly removed, frozen with liquid nitrogen and stored in –80°C freezer. Ovaries were collected from 3-4 rats for each time point. The University of Kansas Animal Care and Use Committee approved protocols for the care and use of rats used in the experiments.

### Regular RT-PCR

Total RNA was extracted from individual ovaries using NucleoSpin® RNA L Kit (Clontech, Palo Alto, CA), with the aide of an Omni Tissue Homogenizer. First-strand cDNA was synthesized from 5 μg of total RNA using ThermoScript (Life Technologies, Carlsbad, CA) in a volume of 40 μl. PCR was done for 30 cycles (denaturation at 94°C for 30 sec, annealing at 59°C for 30 sec, and extension at 72°C for 60 sec) using 1 μl of the first-strand cDNA, 10 pmol of gene specific primers (Table 1) and 2.5 units of JumpStart Taq DNA polymerase (Sigma-Aldrich, St. Louis, MO) in 50 μl of 1X buffer containing 1.5 mM MgCl_2_ and 200 μM dNTP. Aliquots of 18 μl PCR products were analyzed on 2% agarose gels.

**Table 1:** Primers for regular PCR

**Gene**	**Primers**
***Pcsk1***	5′-TATGACCCATTGGCCAATAACC-3′
	5′-TTCCCTTTCAGCCAACAGTACG-3′
***Pcsk2***	5′-CGAAACCAGCTTCACGATGAG-3′
	5′-ACGCCGGCTTAGCAAAATGGA-3′
***Furin***	5′-CTATGGCTACGGGCTGTTGG-3′
	5′-CCTCGCTGGTATTTTCAATCTC-3′
***Pcsk4***	5′-CTTGTGGCCATCAGACCCTTG-3′
	5′-GAACAGGCAGTGTAGTCGCTG-3′
***Pcsk5***	5′-AGTGCGCTCCATCTACAAAGC-3′
	5′-GTCAGTGCAGTGATCCGGTC-3′
***Pcsk6***	5′-TATGGATTTGGCTTGGTGGATG-3′
	5′-GGCTCCATTCTTTCAACTTTCC-3′
***Pcsk7***	5′-CATTGTCTTCACAGCCACTCAG-3′
	5′-CAGTCTGTAGACTCCTCTTGC-3′
***Mbtps1***	5′-TAAACGAGCTGCTGTCTGTGTG-3′
	5′-GAGTAGCGATGAAGGTGGTTTC-3′
***Pcsk9***	5′-GAACTTGGCGTCTCATCCTGG-3′
	5′-CATTGCTTCTCTGGCCCTGTC-3′
***Actb***	5′-GCCAACCGTGAAAAGATGACC-3′
	5′-CCAGACAGCACTGTGTTGGCA-3′
***Rln1***	5′-AGCCAGGAGGAGCCAGCTC-3′
	5′-TCATGACTGAGCATCTGAGCCTAAG-3′

### Real-time PCR

For real-time PCR, cDNA was synthesized from 2 μg of total RNA, using High-Capacity cDNA Reverse Transcription Kit (Life Technologies, Carlsbad, CA). The resulting cDNA was diluted 1:10 with 10 mM Tris HCl, pH 8.0. Real-time PCR was performed with StepOne™ real-time PCR system using the default protocol (Applied Biosystems, Foster City, CA). Each reaction was composed of 10 μl of 2X TaqMan Gene Expression Master Mix, 1 μl of ready-made Gene Expression Assay (Table 2; Applied Biosystems, Foster City, CA), 7 μl of water and 2 μl of diluted cDNA. Reactions were done in triplicate, and the C_T_ values were used to calculate “fold-induction” over Day 0 control using Comparative C_T_ method. ACTB was used as the internal control gene.

**Table 2:** TaqMan gene expression assays from Applied Biosystems

***Gene***	**Catalog number and assay ID**
***Pcsk1***	Cat.#: 4331182, ID: Rn00567266_m1
***Pcsk2***	Cat.#: 4331182, ID: Rn00562543_m1
***Furin***	Cat.#: 4331182, ID: Rn00570970_m1
***Pcsk4***	Cat.#: 4331182, ID: Rn00592006_m1
***Pcsk5***	Cat.#: 4351372, ID: Rn01450817_m1
***Pcsk6***	Cat.#: 4331182, ID: Rn00564475_m1
***Pcsk7***	Cat.#: 4331182, ID: Rn00570376_m1
***Mbtps1***	Cat.#: 4331182, ID: Rn00585707_m1
***Pcsk9***	Cat.#: 4331182, ID: Rn01416753_m1
***Actb***	Cat.#: 4331182, ID: Rn00667869_m1
***Rln1***	Cat.#: 4331182, ID: Rn00566383_m1

### Data analysis

Data points shown represent mean ± standard error. Statistically significant differences between data points of two groups were determined by Student’s t-test. By convention, a *P* value of < 0.05 was considered statistically significant.

## Results

**Figure 1:**
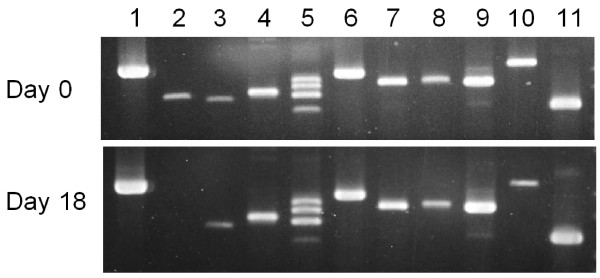
**RT-PCR analyses of proprotein convertase expression in rat ovaries at Day 0 (top panel) and Day 18 (bottom panel) pregnancy using gene-specific primers (Table **1**).** PCR products were analyzed on 2% agarose gel and visualized by ethidium bromide staining. The genes are: *Actb, Pcsk1, Pcsk2, Furin, Pcsk4, Pcsk5, Pcsk6, Pcsk7, Mbtps1, Pcsk9* and *Rln1* (lanes 1-11, respectively).

All nine proprotein convertases are expressed in rat ovaries at Day 0 and Day 18 of pregnancy as determined by RT-PCR (Figure 1), except *Pcsk1* at Day 18 probably due to variation in ovarian sample. In general, the pattern of expression of the nine proprotein convertases in ovaries at Day 18 of pregnancy is similar to that of ovaries at Day 0 of pregnancy. Among the proprotein convertase genes, *Pcsk5* and *Mbtps1* are expressed at the highest expression level; *Furin, Pcsk6, Pcsk7* and *Pcsk9* at a moderate level; whereas *Pcsk1* and *Pcsk2* at the lowest level. With the exception of *Pcsk4*, all proprotein convertases produced single amplicons, which had been confirmed by cloning and nucleotide sequencing (data not shown). The reason for detecting multiple amplicons of *Pcsk4* in ovarian cDNA is unknown, since only one amplicon is detected in testis cDNA using the same set of primers and is confirmed by nucleotide sequencing (data not shown). Redesign of another set of primers generated the same result. The identities of these multiple amplicons were not determined.

**Figure 2:**
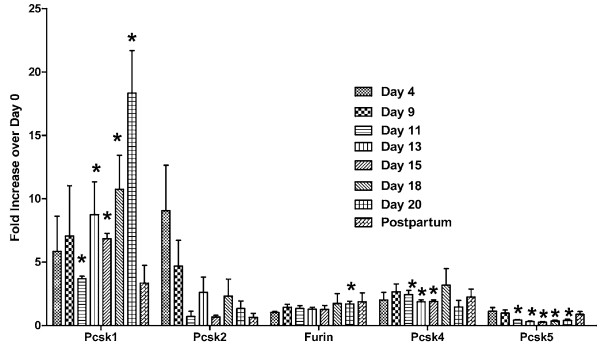
**Relative expression levels of * Pcsk1, Pcsk2, Furin, Pcsk4 *and *Pcsk5 *in rat ovaries at Days 4, 9, 11, 13, 15, 18, and 20 of pregnancy, and 3 days postpartum.** Relative proprotein convertase mRNA levels were determined by real-time PCR analyses as described in Methods. Results were expressed as “Fold Increase over Day 0 control” of the corresponding gene (mean ± S.E.). N = 3; *P < 0.05 compared with Day 0 control.

**Figure 3:**
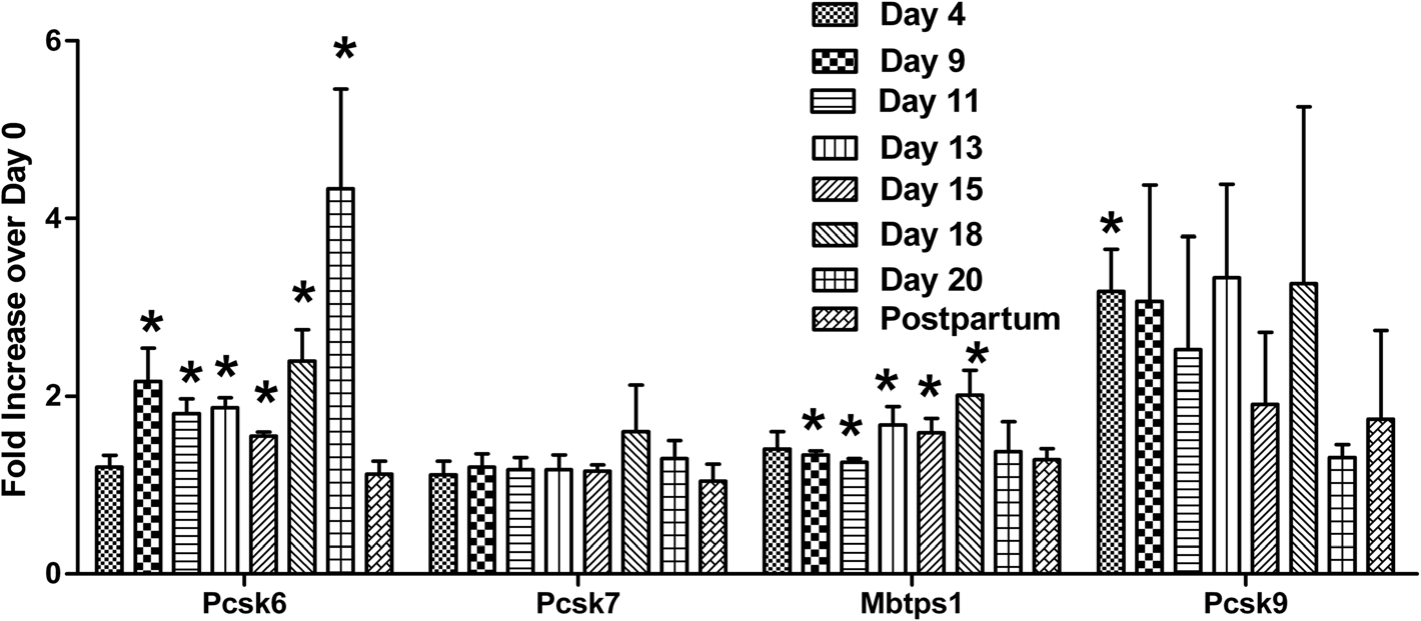
**Relative expression levels of *Pcsk6, Pcsk7, Mbtps1 *and *Pcsk9 *in rat ovaries at Days 4, 9, 11, 13, 15, 18, and 20 of pregnancy, and 3 days postpartum.** Relative proprotein convertase mRNA levels were determined by real-time PCR analyses as described in Methods. Results were expressed as “Fold Increase over Day 0 control” of the corresponding gene (mean ± S.E.). N = 3; *P < 0.05 compared with Day 0 control

**Figure 4:**
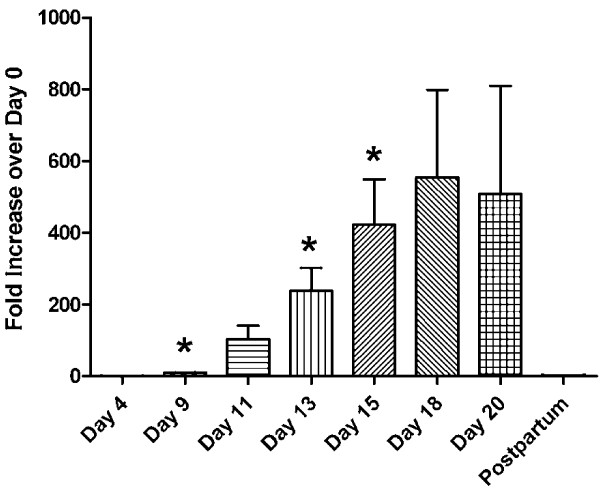
**Relative expression levels of relaxin gene *(Rln1) *in rat ovaries at Days 4, 9, 11, 13, 15, 18, and 20 of pregnancy, and 3 days postpartum.** Relative relaxin mRNA levels were determined by real-time PCR analyses as described in Methods. Results were expressed as “Fold Increase over Day 0 control” of relaxin gene (mean ± S.E.). N = 3; *P < 0.05 compared with Day 0 control.

To determine the relative expression levels of these proprotein convertases at different stages of pregnancy, real-time PCR was used. Relative expression levels of the nine proprotein convertases throughout gestation and at 3 days postpartum are shown in Figures 2 and 3. Four patterns are observed: (1) *Pcsk1* and *Pcsk6* showed upregulation as gestation advanced; (2) *Pcsk2* showed a downregulation trend, although the results were highly variable; (3) *Pcsk5* showed relatively higher levels in early pregnancy and postpartum, but lower level in mid-pregnancy; (4) *Furin, Pcsk4, Pcsk7, Mbtps1 and Pcsk9* showed little change of expression throughout gestation. As a positive control, the expression level of relaxin showed a steady increase as gestation progressed, followed by a precipitous drop at 3 days postpartum (Figure 4).

## Discussion

In the present study, we have determined the relative expression levels of the nine proprotein convertase genes in rat ovaries throughout gestation. Of these nine proprotein convertase genes, only *Pcsk1* and *Pcsk6* were upregulated as gestation advanced, in parallel with the expression level of the relaxin gene. The others did not show a consistent pattern of change in the expression level. The results on the upregulation of *Pcsk1* in rat ovaries during pregnancy agree with those of our previous study using porcine ovary [19]. So far, the relative levels of other proprotein convertases in ovaries during pregnancy have not been reported.

It is difficult to ascribe a specific substrate for each of the proprotein convertases, because of their complementary and redundant functions. Since the levels of *Pcsk1* and *Pcsk6* transcripts were greatly increased in late pregnancy, these enzymes may be involved in the posttranslational processing of prorelaxin, the precursor of the major peptide hormone of the ovary, relaxin, in late pregnancy. Judging from the relative intensities of *Pcsk1* and *Pcsk6* transcripts (Figure 1), PACE4 (PCSK6) may play a more important role in the posttranslational processing of prorelaxin. Although the relative level of *Pcsk1* expression increased over 15-fold at Day 20 of pregnancy while that of *Pcsk6* increased only 4-fold (Figures 2 and 3), *Pcsk6* is expressed at a much higher basal level than *Pcsk1* (Figure 1). Therefore, PCSK6 will be present at a much higher level in the ovary than PCSK1. Whether it plays a more important role than PCSK1 remains to be verified experimentally. It is of great interest to study the posttranslational processing of prorelaxin, since its expression level in the ovary gradually increases during the course of pregnancy. First, results from our laboratory and others showed that prorelaxin is biologically active [20-22]. Unlike the structurally similar proinsulin, proteolytic processing is not necessary for the biological activities of relaxin. Second, with the exception of human and primate prorelaxins [23-25], the paired basic residues required for recognition and cleavage by the first seven members of proprotein convertases are not present at the B-chain/C-peptide junction in prorelaxins of the other species. Processing of these prorelaxins at the B-chain/C-peptide junction will require other proteases. The B-chain/C-peptide junction of rat prorelaxin does not possess the recognition sequence of SKI-1/S1P and PCSK9. Therefore, they are unlikely to be involved in the processing of prorelaxin. The identity of the proteases that process the prorelaxin at the B-chain/C-peptide junction remains to be studied. Third, the processing of prorelaxin is very slow, the majority of relaxin present in rat serum or ovary of late pregnancy were high molecular weight forms. On Day 20 pregnancy, only 25% of relaxin in rat serum was the 6 kDa relaxin [26]. Two forms of high molecular weight (18 and 16.5 kDa) relaxin have also been detected in rat ovaries on Day 20 pregnancy and the combined concentration of these two forms was more than 30 times higher than that of 6 kDa relaxin [27].

As mentioned in the introduction, PC5/6 may be involved in the processing of precursors of transformation growth factor and matrix metalloproteinase family [11], and that of inhibin [12]. On the other hand, furin may be involved in the processing of matrix metalloproteinase 2 [16]. PC4 is suggested to be the enzyme that processes pituitary adenylate cyclase-activating polypeptide [28].

## Conclusions

In conclusion, all nine proprotein convertases are expressed in the rat ovary during gestation. Only PC1/3 and PACE4 were found to be upregulated as gestation advanced. They may play an important role in the processing of proproteins in the ovary during pregnancy. However, it is difficult to ascribe a specific substrate to these proprotein convertases.

## Abbreviations

Actb: β-actin gene; Mbtps1: Membrane-bound transcription factor peptidase, site 1 gene; NARC-1: Neural apoptosis-regulated convertase-1; PACE4: Paired amino acid converting enzyme 4; PC: Proprotein convertase; PCR: Polymerase chain reaction; PCSK: Proprotein convertase subtilisin/kexin; Rln1: Relaxin-1; S1P: Site-1 protease; siRNA: small interfering RNA; SKI-1: Subtilisin/kexin-like isozyme-1; TGFβ: Transforming growth factor beta.

## Competing interests

The authors declare that they have no competing interests.

## Authors’ contributions

SK performed the experiments of molecular biology and wrote the manuscript. DC and MS designed and collect the rat ovaries needed for this study. GD helped to run a preliminary study. All authors read and approved the final manuscript.
